# Establishing the HLS-Q12 short version of the European Health Literacy Survey Questionnaire: latent trait analyses applying Rasch modelling and confirmatory factor analysis

**DOI:** 10.1186/s12913-018-3275-7

**Published:** 2018-06-28

**Authors:** Hanne Søberg Finbråten, Bodil Wilde-Larsson, Gun Nordström, Kjell Sverre Pettersen, Anne Trollvik, Øystein Guttersrud

**Affiliations:** 1 Department of Public Health and Department of Nursing, Faculty of Social and Health Sciences, Inland Norway University of Applied Sciences, PO Box 400, N-2418 Elverum, Norway; 2 Department of Health Sciences, Faculty of Health, Science and Technology, Nursing science, Karlstad University, SE-65188 Karlstad, Sweden; 3 Department of Nursing, Faculty of Social and Health Sciences, Inland Norway University of Applied Sciences, PO Box 400, N-2418 Elverum, Norway; 4 Department of Nursing and Health Promotion, Faculty of Health Sciences, OsloMet – Oslo Metropolitan University, PO Box 4, St Olavs plass, N-0130 Oslo, Norway; 5 Norwegian Centre for Science Education, Faculty of Mathematics and Natural Sciences, University of Oslo, PO Box 1106, Blindern, N-0317 Oslo, Norway

**Keywords:** Confirmatory factor analysis of categorical data, Health literacy, HLS-EU-Q47, HLS-Q12, Rasch modelling, Short version, Validation

## Abstract

**Background:**

The European Health Literacy Survey Questionnaire (HLS-EU-Q47) is widely used in assessing health literacy (HL). There has been some controversy whether the comprehensive HLS-EU-Q47 data, reflecting a conceptual model of four cognitive domains across three health domains (i.e. 12 subscales), fit unidimensional Rasch models. Still, the HLS-EU-Q47 raw score is commonly interpreted as a sufficient statistic. Combining Rasch modelling and confirmatory factor analysis, we reduced the 47 item scale to a parsimonious 12 item scale that meets the assumptions and requirements of objective measurement while offering a clinically feasible HL screening tool. This paper aims at (1) evaluating the psychometric properties of the HLS-EU-Q47 and associated short versions in a large Norwegian sample, and (2) establishing a short version (HLS-Q12) with sufficient psychometric properties.

**Methods:**

Using computer-assisted telephone interviews during November 2014, data were collected from 900 randomly sampled individuals aged 16 and over. The data were analysed using the partial credit parameterization of the unidimensional polytomous Rasch model (PRM) and the ‘between-item’ multidimensional PRM, and by using one-factorial and multi-factorial confirmatory factor analysis (CFA) with categorical variables.

**Results:**

Using likelihood-ratio tests to compare data-model fit for nested models, we found that the observed HLS-EU-Q47 data were more likely under a 12-dimensional Rasch model than under a three- or a one-dimensional Rasch model. Several of the 12 theoretically defined subscales suffered from low reliability owing to few items. Excluding poorly discriminating items, items displaying differential item functioning and redundant items violating the assumption of local independency, a parsimonious 12-item HLS-Q12 scale is suggested. The HLS-Q12 displayed acceptable fit to the unidimensional Rasch model and achieved acceptable goodness-of-fit indexes using CFA.

**Conclusions:**

Unlike the HLS-EU-Q47 data, the parsimonious 12-item version (HLS-Q12) meets the assumptions and the requirements of objective measurement while offering clinically feasible screening without applying advanced psychometric methods on site. To avoid invalid measures of HL using the HLS-EU-Q47, we suggest using the HLS-Q12. Valid measures are particularly important in studies aiming to explain the variance in the latent trait HL, and explore the relation between HL and health outcomes with the purpose of informing policy makers.

## Background

Health literacy (HL) is believed to have a vital impact on public health, including access to and the use of health services [1–3]. Low HL is associated with poorer health conditions [3, 4], more frequent use of health services, longer hospitalisations [2, 3] and higher mortality [4, 5]. Further, some studies relate low HL to unhealthy behaviours, such as smoking [1, 6–9], low physical activity [7–12] and less use of preventive services [1, 4, 7]. Several instruments have been developed to measure individuals’ HL and explore how health outcomes relate to HL. Some instruments assess basic reading and writing skills (such as the Test of Functional HL in Adults [TOFHLA] [13] and the Rapid Estimate of Adult Literacy in Medicine [REALM] [14]), while others assess specific health and cognitive domains (such as the European Health Literacy Survey Questionnaire [HLS-EU-Q47] [11, 15]). The HLS-EU-Q47 was developed from a conceptual model covering four cognitive domains within three health domains, which constitutes a 12-cell matrix for item development [16]. The cognitive domains comprise the proficiency to access (A), understand (B), appraise (C) and apply (D) health information. These competencies are considered necessary to handle health information within health care (HC), disease prevention (DP) and health promotion (HP) settings.

The psychometric properties of the HLS-EU-Q47 have been investigated using principal component analysis (PCA) [15] and confirmatory factor analysis (CFA) [17–19]. When applying CFA in these studies, the health domains were treated as three uncorrelated or orthogonal subscales confirming a four-component structure reflecting the four cognitive domains. A Norwegian study among individuals with type 2 diabetes [20], in which the HLS-EU-Q47 was validated using Rasch-modelling in combination with CFA, revealed that the three health domains were highly correlated and that a 12-dimensional model (reflecting the four cognitive domains across the three health domains in the conceptual framework) obtained the best data-model fit. These results were later confirmed in a Taiwanese study among stroke patients [21]. A 12-dimensional scale requires a multidimensional approach and provides 12 different scores for each individual, which is impractical in clinical settings. Despite the theoretically identified and empirically confirmed multidimensionality of the HLS-EU-Q47, several studies assume a unidimensional latent variable and report one overall HL sum score [3, 9, 11, 17–19, 22–24]. Beside multidimensionality, researchers have observed items with poor fit, redundant items, items displaying unordered response categories and subscales with low precision or reliability [20, 21]. Therefore, we aim to clarify these issues based on a large sample of randomly selected individuals.

A unidimensional short version of the HLS-EU-Q47 will provide clinically useful data in a fast, reliable and accurate manner without applying advanced psychometry. Moreover, measurement scales should be anchored in theory and conceptual frameworks [25]. Hence, it is desirable that the short version reflects the conceptual HL model of Sørensen et al. [16], and that the 12 domains are equally represented. In addition, nurses and other health professionals should be able to use the short version to map patients’ HL without applying advanced psychometric approaches. Several short versions of the HLS-EU-Q47 have been suggested, such as the HLS-EU-Q16 [11] and HL-SF12 [26]. However, we did not find any peer-reviewed publications describing the basis on which the items were selected, whether these short versions were validated using Rasch modelling, or whether they are sufficiently unidimensional. Therefore, based on the findings of Finbråten et al. [20] and the validation procedure in the present study, Rasch modelling and CFA are used to establish a unidimensional 12 item version of the HLS-EU-Q47.

Rasch models meet the assumptions and requirements of fundamental measurement, such as additivity [27], specific objectivity [28], sufficiency [29] and invariance [30]. Both Rasch modelling and CFA can be considered appropriate methods for assessing dimensionality [31, 32]. CFA is used at an overall level to assess discriminant validity (dimensionality) and factorial validity (loadings or common variance), whereas Rasch modelling in addition provide detailed information at the item level, in addition to assessing local independency.

Against this background, the aims of our paper are to: (1) evaluate the psychometric properties of the HLS-EU-Q47 and associated short versions in the Norwegian population and (2) establish a parsimonious unidimensional short version (HLS-Q12) with sufficient psychometric properties. More specifically, we will test the following falsifiable hypothesis: When applied to Norwegian adults, the HLS-EU-Q47 represents a unidimensional, well-targeted scale with acceptable person separation (reliability), consisting of independent and invariant items at the ordinal level (i.e., ordered response categories) each displaying sufficient fit to the partial-credit parameterisation of the unidimensional polytomous Rasch-model.

The same hypothesis is tested for the HLS-Q12, and is used as a basis for comparing the psychometric properties of HLS-Q12 to the HL-SF12 and the HLS-EU-Q16.

## Methods

### Data collection

A random sample of 900 Norwegians aged 16 and over responded to the HLS-EU-Q47 during November 2014. For comparative purposes, we initially aimed at applying identical data collection procedures as the European HL survey [11], but owing to personal resources, it was not possible to perform face-to-face interviews. Hence, data were collected using computer-assisted telephone interviews. The international survey agency, Ipsos, with access to country representative samples, collected the data following detailed instructions offered by the researchers. The European HL survey sampled approximately 1000 respondents in each participating country [11] independent of population size. As there are significantly fewer inhabitants in Norway compared to most of the countries that participated in the European HL survey [33], 900 were sampled. The age limit was set to 16 years as this is the age of licence in relation to health in Norway.

### Measures

The measures included the 47 items HLS-EU-Q47 HL survey questionnaire and additional person factors, such as gender, age and highest level of education completed.

#### The HLS-EU-Q47

Sørensen et al. [11, 15] developed the HLS-EU-Q47 items to reflect their conceptual model, and suggested a 4-point rating scale with response categories ranging from very easy (1) to very difficult (4). In the present study, the rating scale was reversed to make a higher score indicate a higher proficiency at the latent trait. A ‘don’t know’ category, which was later recoded to missing data, was added to record such spontaneous utterings from respondents during the telephone interviews.

#### Translation of the HLS-EU-Q47

The translation procedure, that involved forward and back translation, is thoroughly described in Finbråten et al. [20]. Cognitive interviews were used to explore item interpretation and clarify any ambiguities.

### Data analysis

Rasch modelling was used to assess the psychometric properties of the HLS-EU-Q47 and as a basis for developing the HLS-Q12. Rasch modelling and CFA were used to study the psychometric properties of the short versions HLS-Q12 (our suggested short version), HL-SF12 [26] and the HLS-EU-Q16 [11].

The data were analysed up against the partial-credit parameterisation [34] of the unidimensional polytomous Rasch model (PRM) and the ‘between-item’ multidimensional PRM [35, 36]. Below, the one-dimensional approach refers to unidimensional Rasch analysis. The two- and three-dimensional approaches refer to an oblique or unrestricted multidimensional Rasch analysis where the health domains are allowed to covary. The two-dimensional approach corresponds to the HC subscale and the combined DP and HP subscales, whereas the three-dimensional approach reflects the three health domains. The 12-dimensional approach refers to a similar analysis, where the four cognitive domains and the three health domains define 12 correlated subscales.

The RUMM2030 statistical software package [37] was used for the one-dimensional and consecutive Rasch analyses (treating the subscales as orthogonal or uncorrelated), and the ConQuest 4 statistical software package [38] was used for the one-dimensional and multidimensional Rasch analyses. CFA was performed using LISREL9.3 software [39].

#### Rasch modelling

The null hypothesis of a locally independent scale is weakened and might be rejected in favour of the alternative hypothesis, suggesting a multidimensional scale, in the following situations: When the proportion of individuals with significantly different person-location estimates on a pair of related subscales exceeds 5% (or the lower bound of the binominal 95% confidence interval (CI) exceeds 5%) [40–43]; when the subtest analysis in RUMM points to low common subscale variance and high unique subscale variance (i.e. low fractal index *A* < 0.8 and high fractal index *c* close to 0.5, respectively) [44]; when a multidimensional Rasch model obtains a better data-model fit than a one-dimensional Rasch model (i.e. significantly lower deviance or -2log-likelihoods using a likelihood-ratio test for nested models and AIC for non-nested models) [45]; and/or when absolute values of Rasch-model residual correlations among items exceed 0.3 [46].

Likelihood-ratio tests (LRT) were used to compare data-model fit for nested models, such as the one-, two-, three- and 12-dimensional Rasch models, where the first implies parallel subscales (correlations between all theoretically defined subscales were fixed at 1) and the latter implies freely correlated subscales. The LRT statistic “difference in deviance” is asymptotically *χ*2 distributed with degrees of freedom (*df*) equal to the difference in the number of estimated model parameters comparing two nested models [47]. Akaike’s information criterion (AIC) [48] was used to compare the data-model fit for non-nested models, such as the various short versions. Similar to Allen and Wilson [49], both deviance and AIC are reported when applying the consecutive approach to the HLS-EU-Q47 (calculated by adding the values for each of the three subscales). Lower values of deviance and AIC indicate better data-model fit. In addition to deviance and AIC, total item chi-square with *p*-values was used to assess the data-model fit of the short versions.

Items were interpreted as under−/over-discriminating relative to the Rasch model when the infit MNSQ were significantly different from the expected value of 1 (indicating perfect fit to model) or, more precisely, when the infit value was above/below the 95% CI and the corresponding *t* value was > 1.96 [45, 50].

In addition to infit, chi-square probability was used to assess item fit for the various short versions. Items were considered as misfitting at high chi-square values and probability values lower than a Bonferroni-adjusted 5% [51]. Item difficulty was described using item-location estimates.

Applying the consecutive approach to the HLS-EU-Q47 and the one-dimensional approach to the short versions, differential item functioning (DIF) was explored using analysis of variance (ANOVA) [40] for the dichotomized person factors of gender, age and level of highest completed education: age was dichotomised as above or below the sample mean (47 years), and highest level of completed education was split into “compulsory and upper secondary school” versus “university level”.

The ordering of the response categories was examined applying the one-dimensional and the consecutive approaches. Significantly different thresholds in “correct” order [52, 53] were used as evidence for ordered response categories. The targeting of the (sub)scales was evaluated by comparing the distribution of the item threshold estimates to the distribution of the person estimates [51]. For more detailed descriptions of the analyses performed, see for example, Andrich [30], Hagquist [53], Hagquist, Bruce and Gustavsson [54] or Finbråten et al. [20].

#### Confirmatory factor analysis

The null hypothesis of a one-dimensional scale is weakened when overall fit indexes for multifactorial CFA are superior to a one-factorial CFA [55]. Owing to categorical raw data, we defined the variables as ordinal in the “lsf” file and estimated the polychoric item correlations and their asymptotic covariance matrix using the PRELIS application to LISREL [56]. As target values for goodness-of-fit (GOF) indexes is based on maximum likelihood estimation (ML), we used robust ML to obtain the Satorra-Bentler scaled chi-square GOF index (SB scaled χ^2^) [57, 58]. The following overall GOF indexes for model comparison based on the null model were used: SB scaled χ^2^, standardized root mean square residual (SRMR), root mean square error of approximation (RMSEA), comparative fit index (CFI) and non-normed fit index (NNFI, or Tucker-Lewis index [TLI]). Schumacker and Lomax [59] recommend SRMR < .05, RMSEA ≤ .05, and CFI and NNFI ≥ .95 as GOF index target values, whereas Hu and Bentler [60] claim that SRMR and RMSEA values close to or below 0.8 and 0.6, respectively, indicate sufficient overall fit. The AIC and the Bayesian information criterion (BIC) were also taken into account. AIC and GOF indexes were used to compare the various short versions and to compare the one-factor to the three-factor approaches for the various short versions. Item communalities (squared factor loadings for completely standardized solution) describe the shared variance among the items that is accounted for by the latent variable, HL [61].

#### Reliability

Person separation index (PSI), person separation reliability (PSR) and the *H* coefficient [62] (obtained from RUMM, ConQuest and calculated manually based on communalities from CFA) were used as measures of reliability. Values exceeding 0.85 (0.65) are sufficient if conclusions are to be drawn at the individual (group) level [63].

### Developing a one-dimensional short version

The suggested short version in the present study, the HLS-Q12, was developed from analyses of the HLS-EU-Q47 in two Norwegian samples; people with type 2 diabetes [20] and the general population. The suggested version, HLS-Q12, was developed by stepwise exclusion of poorly fitting items, items displaying DIF and items collecting redundant information, together with a qualitative evaluation of the item content in light of the conceptual framework. For dependent items, the item with the most essential information was retained, while the other item was discarded. We ensured that the items in our final scale (specified in Table 5) were distributed across the HLS-EU 12-cell matrix [11, 16].

### Missing data

The dataset we analysed was complete, but there was, on average, 29 (3%) ‘don’t know’ responses per item, which we recoded as missing values. Item 2 had the highest number of ‘don’t know’ responses (69 or 8%). Rasch modelling was performed on incomplete data (except in the subtest analyses), whereas LISREL used list-wise deletion as the default method, reducing the sample size from 900 to effective sample sizes of 699, 680 and 670 for the HLS-Q12, HL-SF12 and the HLS-EU-Q16, respectively.

## Results

The sample (*n* = 900) distributions for gender and age reflected the population distributions, while individuals with higher education were somewhat over-sampled (Table 1).

**Table 1:** Characteristics of study sample and the Norwegian population

	Sample *(n = 900)*	Population^a^ *(N = 4,115,195)*
Age		
mean ± sd	47.0 ± 17.7	46.5 ± 19.0
- median	48	45
- min−max	16−91	16−105
- missing	0	0
	%	%
16–24 years	13	15
25–39 years	25	25
40–54 years	25	26
55–66 years	21	17
67–79 years	14	12
80+ years	2	5
Gender	*n* (%)	*n* (%)
- males	441 (49)	2,567,434 (50)
- females	459 (51)	2,541,622 (50)
- missing	0	
Highest completed education	*n* (%)	%
- compulsory comprehensive school	87 (10)	26
- upper-secondary school	298 (33)	38
- university level, lower degree^b^	321 (36)	23
- university level, higher degree^c^	191 (21)	9
- missing	3 (< 1)	

^a^Norwegian population 16 years and over in 2014 (Statistics Norway, 2017)

^b^Education at university or university college for 4 years or less (Bachelor’s degree)

^c^Education at university or university college for 5 years or more (Master’s degree and/or PhD)

### Psychometric properties of the HLS-EU-Q47

#### Overall analyses of HLS-EU-Q47

Overall analyses included those for dimensionality, data-model fit, response dependence and reliability. The initial analyses of dimensionality indicated that neither the entire HLS-EU-Q47 nor the three health domains, HC, DP and HP, were sufficiently unidimensional (the respective proportions of significant *t*-tests were 21, 9, 13 and 10% [not reported in Table 2]).

**Fig. 1:**
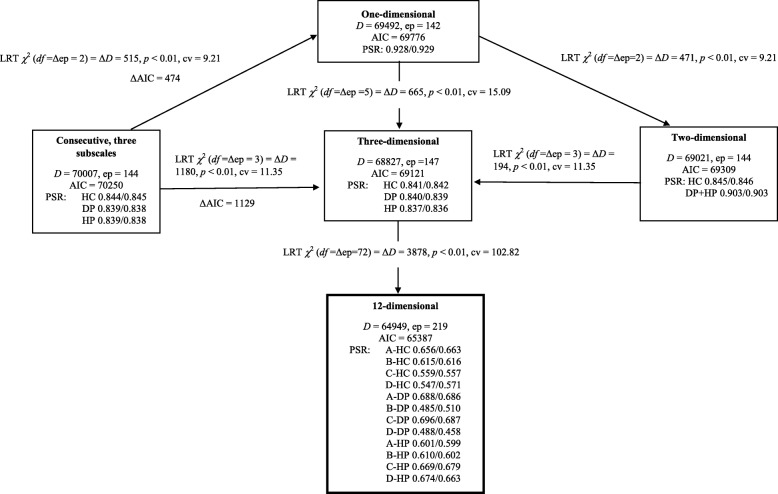
Model fit of the HLS-EU-Q47 after applying various analysis approaches. Figure 1 shows the overall fit statistics for the one-dimensional approach (all subscale correlations fixed to 1), the consecutive approach (treating the three health domains as orthogonal or uncorrelated) and the two-, three- and 12-dimensional approaches (treating the theoretical subscales as correlated). A: access, B: understand, C: appraise, D: apply (cognitive domains). HC: health care, DP: disease prevention, HP: health promotion (health domains). Δ: change in parameter, AIC: Akaike’s information criterion, cv: critical value, *D*: deviance, *df*: degrees of freedom, ep: number of estimated parameters, LRT: likelihood ratio test. PSR: person separation reliability based on marginal maximum likelihood estimate/Warm’s mean weighted likelihood estimate

Using LRT, the observed data were more likely under a 12-dimensional Rasch model than a one-, two- or three-dimensional Rasch model (Fig. 1). As expected, the data-model fit improved significantly in each step from the one-dimensional through the 12-dimensional model. For example, the drop in deviance from the three- to the 12-dimensional model was Δ*D* = 3878 ≈ *χ*^*2*^ [Δ*df* = Δestimated parameters (ep) = 72], *p* < 0.01, critical value = 103. The three-dimensional model had a lower AIC and a better fit than the consecutive approach of the three health domains, HC, DP and HP (ΔAIC = 1129). The correlations between HC and DP, HC and HP, and DP and HP were *r* = 0.81, 0.71 and 0.83, respectively.

Applying the one-dimensional approach, we observed nine pairs of dependent items (1 and 2, 22 and 23, 41 and 42, 44 and 46, 45 and 47, 3 and 7, 12 and 28, 17 and 21, 30 and 37), of which the latter four pairs surprisingly appeared *across* the theoretically defined subscales. Applying the consecutive approach to the three health domains, only one pair of dependent items was observed within each health domain (1 and 2, 17 and 21, and 41 and 42, respectively).

Applying the 12-dimensional approach to the HLS-EU-Q47, we observed relatively low subscale reliability indexes, most likely owing to few items per scale—on average 3.9 items per subscale (see PSR values in Fig. 1). Consequently, we observed acceptable reliability indexes for the one-dimensional approach and for the subscales of the two- and the three-dimensional approaches.

#### Analyses of HLS-EU-Q47 at item level

Analyses at the item level included analyses of item fit, item discrimination, DIF and ordering of response categories. Table 2 shows the item fit when applying the one-, three- and 12-dimensional approaches.

**Table 2:** Single-item characteristics of the HLS-EU-Q47 given various analytical approaches

			One-dimensional approach						Three-dimensional approach				Consecutive three-dimensional approach		12-dimensional approach				Consecutive 12-dimensional approach
HD	CD	Item	Infit	CI		*t*	Ordered	DIF	Infit	CI		*t*	Ordered	DIF	Infit	CI		*t*	DIF
		On a scale from very difficult to very easy, how easy would you say it is to:																	
HC	A	1) find information about symptoms of illnesses that concern you?	1.01	0.90	1.10	0.3		Age, y > o	0.98	0.90	1.10	−0.4		Age, y > o	1.03	0.89	1.11	0.6	
“	A	2) find information on treatments of illnesses that concern you?	0.99	0.90	1.10	−0.3			0.95	0.90	1.10	−1.0			0.99	0.90	1.10	−0.2	
“	A	3) find out what to do in case of a medical emergency?	1.02	0.91	1.09	0.3			0.97	0.91	1.09	−0.7			1.07	0.90	1.10	1.4	
“	A	4) find out where to get professional help when you are ill?	0.99	0.89	1.11	−0.1			0.96	0.89	1.11	−0.8			1.06	0.89	1.11	1.1	
“	B	5) understand what your doctor says to you?	0.96	0.90	1.10	−0.7		Edu, h > l	0.91	0.90	1.10	−1.7		Edu, h > l	0.95	0.90	1.10	−0.9	Edu, h > l
“	B	6) understand the leaflets that come with your medicine?	1.06	0.90	1.10	1.3			1.04	0.90	1.10	0.9			1.03	0.90	1.10	0.6	
“	B	7) understand what to do in a medical emergency?	1.00	0.91	1.09	0.1			0.96	0.91	1.09	−0.9			0.99	0.91	1.09	−0.1	Edu, h < l
“	B	8) understand your doctor’s or pharmacist’s instruction on how to take a prescribed medicine?	0.93	0.89	1.11	−1.3	No		0.89	0.89	1.11	−2.0^b^	No		0.92	0.88	1.12	−1.3	
“	C	9) judge how information from your doctor applies to you?	0.96	0.89	1.11	−0.6			0.94	0.89	1.11	−1.1			0.96	0.89	1.11	−0.7	
“	C	10) judge the advantages and disadvantages of different treatment options?	0.96	0.90	1.10	−0.8			0.99	0.90	1.10	−0.2			0.92	0.90	1.10	−1.7	
“	C	11) judge when you may need to get a second opinion from another doctor?	1.09	0.91	1.09	1.8		Age, y < o	1.14	0.90	1.10	2.7^a^		Age, y < o Edu, h < l	1.06	0.90	1.10	1.1	Age, y < o
“	C	12) judge if the information about illness in the media is reliable?	1.16	0.90	1.10	3.1^a^			1.25	0.90	1.10	4.7^a^			1.10	0.90	1.10	2.0^a^	
“	D	13) use information the doctor gives you to make decisions about your illness?	0.92	0.90	1.10	−1.6		Gen, m > f	0.93	0.90	1.10	−1.4		Gen, m > f	0.93	0.90	1.10	−1.5	Age, y < o
“	D	14) follow the instructions on medication?	0.98	0.88	1.12	−0.3			0.97	0.88	1.12	−0.5			0.90	0.87	1.13	−1.6	
“	D	15) call an ambulance in an emergency?	1.07	0.90	1.10	1.4	No	Gen, m > f Age, y > o	1.07	0.90	1.10	1.4	No	Gen, m > f Age, y > o	0.96	0.89	1.11	−0.7	Age, y > o
“	D	16) follow instructions from your doctor or pharmacist?	0.88	0.89	1.11	−2.1^b^			0.89	0.89	1.11	−1.9			0.81	0.87	1.13	−2.9^b^	
DP	A	17) find information about how to manage unhealthy behaviour such as smoking, low physical activity and drinking too much?	0.93	0.90	1.10	−1.3		Edu, h > l	0.96	0.90	1.10	−0.7		Edu, h > l	1.06	0.90	1.10	1.2	Edu, h > l
“	A	18) find information on how to manage mental health problems like stress or depression?	0.95	0.91	1.09	−1.1			1.00	0.91	1.09	0.0			1.18	0.91	1.09	3.5^a^	
“	A	19) find information about vaccinations and health screenings that you should have?	0.89	0.91	1.09	−2.3^b^			0.93	0.91	1.09	−1.4			1.13	0.91	1.09	2.6^a^	
“	A	20) find information on how to prevent or manage conditions like being overweight, high blood pressure or high cholesterol?	0.88	0.89	1.11	−2.2^b^			0.91	0.89	1.11	−1.6			0.96	0.89	1.11	−0.7	
“	B	21) understand health warnings about behaviour such as smoking, low physical activity and drinking too much?	0.94	0.89	1.11	−1.2	No	Edu, h > l	0.94	0.90	1.10	−1.1	No	Age, y > o Edu, h > l	0.90	0.89	1.11	−1.7	
“	B	22) understand why you need vaccinations?	1.01	0.90	1.10	0.3		Age, y > o	1.03	0.90	1.10	0.6		Age, y > o	1.01	0.90	1.10	0.1	Edu^c^
“	B	23) understand why you need health screenings?	0.94	0.89	1.11	−1.0			0.97	0.89	1.11	−0.5		Age, y > o	0.91	0.89	1.11	−1.5	
“	C	24) judge how reliable health warnings are, such as smoking, low physical activity and drinking too much?	0.99	0.90	1.10	−0.1			1.01	0.90	1.10	0.2			1.06	0.90	1.10	1.2	Age, y > o
“	C	25) judge when you need to go to a doctor for a check-up?	0.98	0.91	1.09	−0.4		Age, y < o	1.03	0.91	1.09	0.7		Age, y < o Edu, h < l	1.00	0.91	1.09	0.1	Age, y < o Edu, h < l
“	C	26) judge which vaccinations you may need?	1.03	0.91	1.09	0.7			1.05	0.91	1.09	1.0			1.06	0.91	1.09	1.2	
“	C	27) judge which health screenings you should have?	0.92	0.91	1.09	−1.9		Age, y < o	0.94	0.91	1.09	−1.4		Age, y < o	0.92	0.91	1.09	−1.8	Age, y < o
“	C	28) judge if the information on health risks in the media is reliable?	1.03	0.90	1.10	0.6			1.05	0.90	1.10	1.0			1.02	0.90	1.10	0.4	Age, y > o
“	D	29) decide if you should have a flu vaccination?	1.18	0.91	1.09	3.6^a^		Age, y < o	1.24	0.91	1.09	4.7^a^		Age, y < o	1.06	0.91	1.09	1.3	Age, y < o
“	D	30) decide how you can protect yourself from illness based on advice from family and friends?	1.06	0.91	1.09	1.3			1.12	0.91	1.09	2.5^a^		Edu, h < l	1.00	0.91	1.09	0.0	Age, y > o Edu, h < l
“	D	31) decide how you can protect yourself from illness based on information in the media?	0.98	0.91	1.09	−0.3			1.01	0.91	1.09	0.3			0.93	0.91	1.09	−1.5	
HP	A	32) find information on healthy activities such as exercise, healthy food and nutrition?	0.96	0.90	1.10	−0.7			0.96	0.90	1.10	−0.8			0.95	0.90	1.10	−1.0	
“	A	33) find out about activities that are good for your mental well-being?	0.97	0.90	1.10	−0.6			0.93	0.90	1.10	−1.3			0.93	0.90	1.10	−1.5	Gen, m < f
“	A	34) find information on how your neighbourhood could be more health-friendly?	1.04	0.90	1.10	0.8			1.01	0.90	1.10	0.2			0.98	0.90	1.10	−0.4	
“	A	35) find out about political changes that may affect health?	1.10	0.90	1.10	1.8			1.08	0.90	1.10	1.5			1.06	0.89	1.11	1.0	
“	A	36) find out about efforts to promote your health at work?	1.02	0.91	1.09	0.3			1.02	0.91	1.09	0.5			1.02	0.91	1.09	0.3	
“	B	37) understand advice on health from family members or friends?	1.06	0.90	1.10	1.2		Age, y > o	1.07	0.90	1.10	1.4		Age, y > o	1.06	0.90	1.10	1.1	Age, y > o
“	B	38) understand information on food packaging?	1.12	0.91	1.09	2.5^a^			1.16	0.91	1.09	3.3^a^		Age, y > o	1.12	0.91	1.09	2.5^a^	Gen, m > f
“	B	39) understand information in the media on how to get healthier?	0.97	0.90	1.10	−0.7			0.97	0.90	1.10	−0.7			0.96	0.90	1.10	−0.8	
“	B	40) understand information on how to keep your mind healthy?	0.91	0.91	1.09	−2.0^b^			0.89	0.91	1.09	−2.3^b^			0.93	0.91	1.09	−1.4	Age, y < o
“	C	41) judge how where you live affects your health and well-being?	1.01	0.90	1.10	0.3		Age, y < o	0.97	0.90	1.10	−0.5		Age, y < o	1.02	0.9	1.10	0.4	
“	C	42) judge how your housing conditions help you to stay healthy?	0.99	0.90	1.10	−0.2		Age, y < o	0.96	0.90	1.10	−0.9		Age, y < o	0.99	0.9	1.10	−0.3	Edu, h < l
“	C	43) judge which everyday behaviour is related to health?	0.92	0.89	1.11	−1.5			0.90	0.89	1.11	−1.8			1.15	0.89	1.11	2.5^a^	Age, y > o Edu, h > l
“	D	44) make decisions to improve your health?	1.06	0.91	1.09	1.3			1.00	0.91	1.09	0.0			0.99	0.91	1.09	−0.1	
“	D	45) join a sports club or exercise class if you want to?	1.12	0.90	1.10	2.3^a^			1.06	0.90	1.10	1.3			1.01	0.90	1.10	0.2	
“	D	46) influence your living conditions that affect your health and well-being?	1.05	0.91	1.09	1.0			0.98	0.91	1.09	−0.3			1.01	0.91	1.09	0.2	Age, y > o
“	D	47) take part in activities that improve health and well-being in your community?	1.10	0.91	1.09	2.0^a^			1.04	0.91	1.09	0.8			1.04	0.90	1.10	0.9	

*Note*. This table reports the item-fit indexes after applying the one-dimensional (subscale correlation fixed to 1), three-dimensional and 12-dimensional approaches (treating the subscales as correlated). The analyses of the ordering of response categories (ordered) and differential item functioning (DIF) are based on the one-dimensional and consecutive approaches (treating the subscales as orthogonal or uncorrelated) for the three health domains and the 12 subscales. Ordered: “No” refers to an item with unordered response categories

^a^A *t*–value > 1.96 and an infit-value > 1 indicate a poor fit with the Rasch model due to there being *more* variation in the data than was expected by the model (item is under-discriminating)

^b^A *t*–value <−1.96 and an infit-value < 1 indicate a poor fit with the Rasch model due to there being *less* variation in the data than was expected by the model (item is over-discriminating)

For items displaying uniform DIF, the relevant dichotomized person factor levels are indicated. For example, “Age, y > o” refers to a uniform DIF for the person factor age in favour of the y = younger respondents (47 years or younger) factor level compared to the o = older respondents (48 years or older) factor level. The “Highest completed education level” (Edu) factor has l = low (primary and secondary school) and h = high (university or university college) levels, and the “Gender” factor (Gen) has the f = females and m = males levels

^c^non-uniform DIF (item 22). All other DIFs were uniform

A: access, B: understand, C: appraise, CD: cognitive domain, CI: confidence interval, D: apply, DP: disease prevention, HC: healthcare, HD: health domain, HP: health promotion

RUMM2030 was used for the analyses of the ordering of response categories and DIF. ConQuest 4 was used for all other analyses

Under-discriminating items were observed using the one- (items 12, 29, 38, 45 and 47), three- (items 11, 12, 29, 30 and 38) and 12-dimensional (items 12, 18, 19, 38 and 43) approaches; items 12 and 38 under-discriminated in all analysis approaches. Over-discriminating items were also observed when the one- (items 16, 19, 20 and 40), three- (items 8 and 40) and 12- dimensional (item 16) approaches were applied. Using the consecutive approach, several items displayed DIF (Table 2).

Unordered response categories were observed for items 8, 15 and 21 when applying the one-dimensional approach, as well as when analysing the HC, DP and HC health domains consecutively (Table 2).

### Psychometric properties of the HLS-EU-Q47 short versions HLS-Q12, HL-SF12 and HLS-EU-Q16

#### Overall analyses of the HLS-Q12, HL-SF12 and HLS-EU-Q16

According to the *t*-tests, none of the short versions could be deemed sufficiently unidimensional (the proportions of significant *t*-tests were above 5% and the lower binominal 95% CI proportion was 0.07 for both the HLS-Q12 and the HL-SF12, whereas the lower binominal 95% CI-proportion was 0.09 for the HLS-EU-Q16 (Table 3).

**Table 3:** Unidimensionality, data-model fit and reliability applying Rasch modelling of the various short versions

	HLS-Q12^a^		HL-SF12 [26]		HLS-EU-Q16 [11]	
	One-dimensional	Three-dimensional	One-dimensional	Three-dimensional	One-dimensional	Three-dimensional
Unidimensionality						
Proportion (%) of significant *t*-tests (lower CI-prop)	8.78% (0.07)		8.01% (0.07)		10.44% (0.09)	
PSI	0.767		0.759		0.830	
PSI^b^	0.687		0.668		0.703	
Fractal indexes		na		na		na
*c*	0.50		0.39		0.46	
*A*	0.90		0.91		0.85	
*r*	0.80		0.87		0.82	
Total item chi square (*df*), probability	112.61 (108), 0.361		130.21 (108), 0.072		208.90 (144), 0.00034	
Log-likelihoods						
Deviance (ep)	18,590 (37)	18,556 (42)	18,707 (37)	18,696 (42)	22,964 (49)	22,815 (54)
AIC (ep)	18,664 (37)	18,640 (42)	18,781 (37)	18,780 (42)	23,062 (49)	22,923 (54)
Reliability						
PSR (MLE)	0.762	0.537/0.517/0.575	0.758	0.501/0.497/0.564	0.825	0.713/0.619/0.545

*Note.* The table shows the results of the tests of unidimensionality based on paired *t*-tests of person-location estimates for subsets of items. It also reports fractal indexes, *c*, *A* and *r*. The person separation index (PSI) and fractal indexes were estimated for the complete dataset (HLS-Q12 *n* = 696, HL-SF12 *n* = 680, HLS-EU-Q47 *n* = 670). Significant *t*-tests ≤5% (or lower confidence interval [CI] proportion ≤ 5%), small drops in PSI (^b^ after adjusting for violations of local independence due to subtest structure), high values of *A* and *r,* and low values of *c* could indicate unidimensionality. Analyses were performed by using RUMM2030 software

Log-likelihoods and person separation reliability (PSR) were estimated for the one- and three-dimensional approaches to the short versions by using ConQuest4 software. Lower values of deviance and AIC indicate a better fit

AIC: Akaike’s information criterion, ep: number of estimated parameters, na: not applicable, PSR (MLE): person separation reliability based on a marginal maximum likelihood estimate

All the short versions are developed on the basis of the HLS-EU-Q47

^a^HLS-Q12 developed through the present study

Nevertheless, the fractal indexes indicated that the HLS-Q12 and HL-SF12 had relatively high amounts of common variance (*A* = 0.90 and 0.91, respectively), high subscale correlation (*r* = 0.80 and 0.87, respectively), and relatively high unique subscale variance (*c* = 0.50 and 0.39, respectively).

Using LRT, the data fit better to the three-dimensional models compared to the one-dimensional models of the various short versions (three-dimensional models had significantly lower deviance, except for the HL-SF12, where the difference was insignificant). The GOF indexes obtained via CFA were also better for the three-factor models as compared to the one-factor models of the various short versions (Table 4).

**Table 4:** Overall fit and reliability using confirmatory factor analyses of the various short versions

	HLS-Q12^a^		HL-SF12 [26]		HLS-EU-Q16 [11]	
GOF index (LISREL)	One-dimensional	Three-dimensional	One-dimensional	Three-dimensional	One-dimensional	Three-dimensional
SB scaled χ^2^ (*df*), χ^2^ *p-value*	142.71 (54), *p* < 0.001	112.96 (51) *p* < 0.001	93.73 (54), *p* < 0.001	83.71 (51) *p* < 0.01	379.12 (104), *p* < 0.001	283.57 (101) *p* < 0.001
SRMR	0.059	0.056	0.047	0.045	0.080	0.070
RMSEA (90% CI)	0.086 (0.078–0.095)	0.078 (0.069–0.087)	0.066 (0.057–0.076)	0.064 (0.055–0.074)	0.118 (0.112–0.124)	0.103 (0.097–0.110)
CFI	0.951	0.966	0.975	0.979	0.923	0.949
NNFI (TLI)	0.940	0.955	0.969	0.973	0.911	0.939
Log-likelihoods						
-2ln(L)(ep)	6857 (24)	6787 (27)	6718 (24)	6696 (27)	8105 (32)	7851 (35)
AIC (ep)	6905 (24)	6841 (27)	6766 (24)	6750 (27)	8169 (32)	7921 (35)
BIC (ep)	7014 (24)	6964 (27)	6874 (24)	6872 (27)	8313 (32)	8078 (35)
Reliability						
*Coefficient H*	0.826	0.663/0.608/0.713	0.822	0.591/0.599/0.622	0.882	0.823/0.720/0.667

*Note.* The table shows goodness of fit (GOF) indexes, log-likelihood and reliability using confirmatory factor analysis (CFA) when treating the various short versions as one- and three-dimensional. CFA was performed using LISREL software

SB scaled χ^2^ :Satorra-Bentler scaled chi-square. SRMR (standardised root mean square residual) and RMSEA (root-mean-squared error of approximation): values < 0.05 indicate good model fit, and values < 0.08 indicate an acceptable model fit. CFI (comparative fit index) and NNFI (non-normed fit index [or TLI = Tucker and Lewis fit index]): values > 0.95 indicate good model fit (Hu and Bentler, 1999)

-2ln(L): likelihood function, AIC: Akaike’s information criterion, BIC: Bayesian information criterion, ep: number of estimated parameters. Lower values indicate a better overall fit

^a^HLS-Q12 developed through the present study

Comparing the short versions, the HLS-Q12 obtained best fit to the Rasch model (had the lowest total item chi-square value and the lowest AIC; Table 3) whereas the HL-SF12 obtained best fit when performing CFA (had the lowest likelihood estimates and the best GOF indexes). The HLS-Q12 also had acceptable GOF indexes (Table 4). The HLS-EU-Q16 displayed the weakest overall fit indexes (highest total item *χ*^2^ value and highest log likelihood estimates).

All the one-dimensional short versions had acceptable reliability values (PSI and PSR values above 0.75 and the *H* coefficient was above 0.82). The HLS-EU-Q16 had the highest reliability indexes (PSI = 0.830, PSR = 0.826 and H = 0.882). The reliability indexes were slightly higher for the HLS-Q12 than for the HL-SF12 (Tables 3 and 4). Applying the three-dimensional approach, the PSR and *H* values of the correlated subscales were below the recommended values (Tables 3 and 4). The HLS-Q12 was the best-targeted, with a mean person location of 0.759. The mean person locations for the HL-SF12 and HLS-EU-Q16 were 0.816 and 1.020, respectively (not shown in the table).

#### Analyses of HLS-Q12, HL-SF12 and HLS-EU-Q16 at the item level

Compared with the HL-SF12 and the HLS-EU-Q16, the HLS-Q12 showed best fit at the item level as there were no occurrences of misfitting items, items displaying DIF with regard to available person factors or items with unordered response categories. For the HL-SF12, items were within-item biased (items 15, 26 and 30 displayed DIF), and one item with unordered response categories was observed (item 15). Problems with regard to DIF (items 5, 11, 13, 21 and 37) and unordered response categories (items 8 and 21) were also observed for the HLS-EU-Q16. In addition, item 11 under-discriminated and showed significant misfit (chi-square probability = 0.001; Table 5). A high level of unique variance was observed for the items of the various short versions (relatively low communality values).

**Table 5:** Item-fit indexes applying the one-dimensional approach to the various short versions

	HLS-Q12^b^					HL-SF12 [26]							HLS-EU-Q16 [11]						
Item	Loc.	Chi prob.	Infit	*t*	Com.	Loc	Chi prob.	DIF	ordered	Infit	*t*	Com.	Loc	Chi prob.	DIF	Ordered	Infit	*t*	Com.
1																			
2	−0.097	0.997	1.01	0.2	0.269	− 0.041	0.699			0.99	−0.1	0.278	0.133	0.606			1.03	0.5	0.276
3																			
4													−0.236	0.703			1.00	0.0	0.338
5													−0.453	0.313	Edu, h > l		0.97	−0.7	0.387
6						0.137	0.348			1.05	1.0	0.183							
7	0.053	0.705	1.03	0.7	0.239														
8													−0.865	0.087		No	0.94	−1.1	0.376
9																			
10	0.892	0.761	0.97	−0.6	0.292	0.964	0.848			1.00	−0.1	0.266							
11													1.084	0.001*	Age, y < o Edu, h < l		1.17	3.4^a^	0.139
12																			
13													−0.082	0.332	Gen m > f		0.97	−0.6	0.319
14	−0.625	0.275	1.00	0.0	0.260														
15						−0.842	0.025	Gen, m > f Age, y > o	No	1.06	1.2	0.207							
16													−1.305	0.044			0.91	−1.6	0.443
17																			
18	0.420	0.357	0.98	−0.5	0.363	0.490	0.006			0.93	−1.6	0.416	0.687	0.633			1.00	0.1	0.299
19																			
20																			
21													−0.793	0.002*	Edu, h > l	No	0.95	−0.9	0.347
22																			
23	−1.158	0.065	0.98	−0.3	0.281	−0.997	0.431			0.96	−0.7	0.291	−0.908	0.385			0.96	−0.7	0.319
24																			
25																			
26						0.526	0.129	Age, y < o		1.07	1.5	0.187							
27																			
28	1.068	0.265	1.04	0.9	0.176								1.360	0.039			1.07	1.4	0.232
29																			
30	0.422	0.284	1.03	0.6	0.230	0.502	0.845	Edu, h < l		1.04	0.9	0.217							
31													1.167	0.166			0.98	−0.4	0.349
32	−1.135	0.618	0.98	−0.5	0.337														
33						−0.298	0.652			0.97	−0.5	0.321	−0.110	0.820			1.03	0.6	0.240
34																			
35																			
36																			
37													0.242	0.070	Age, y > o		1.06	1.2	0.220
38	0.702	0.484	1.06	1.3	0.196														
39						0.372	0.831			1.00	0.0	0.293	0.531	0.305			0.96	−0.7	0.366
40																			
41																			
42																			
43	−0.728	0.375	0.95	−0.9	0.426	−0.621	0.236			0.94	−1.1	0.385	−0.452	0.450			1.00	0.0	0.322
44	0.186	0.105	1.04	0.8	0.248														
45						−0.194	0.072			1.07	1.4	0.202							
46																			
47																			

*Note.* This table shows the item-fit indexes when applying the one-dimensional approach to the various short versions. The HLS-EU-Q16 could not be deemed sufficiently unidimensional. The results are displayed for comparison purposes

Chi square probability (Chi prob), differential item functioning (DIF), item location estimate (Loc) and unordered response categories (ordered) were obtained from Rasch modelling using RUMM2030 software. ConQuest 4 software was used for the Infit measures

^a^A *t*–value > 1.96 and an infit-value > 1 indicate a poor fit with the Rasch model due to there being *more* variation in the data than expected given the model (item is under-discriminating)

*Item with a significant misfit (*p*-value below Bonferroni-adjusted 5%)

For items displaying uniform DIF, the relevant dichotomized person factor levels are indicated. For example, “Age, y > o” refers to the uniform DIF for the person factor age in favour of the y = younger respondents (47 years or younger) factor level as compared to o = older respondents (48 years or older) factor level. The “Highest completed education level” (Edu) factor has the l = low (primary and secondary school) and h = high (university or university college) levels, and the factor “Gender” (Gen) has the f = females and m = males levels

Ordered: “No” refers to items with unordered response categories

None of the items on the HLS-Q12 displayed DIF or unordered response categories

Com: communalities obtained from CFA using the LISREL software

^b^HLS-Q12 developed through the present study

Investigating the item-location estimates of the HLS-Q12, items 28 (*judge if the information on health risks in the media is reliable*) and 10 (*judge the advantages and disadvantages of different treatment options*) had the highest location estimates (1.068 and 0.892, respectively) and were thus the hardest to endorse. Items 23 (*understand why you need health screenings*) and 32 (*find information on healthy activities such as exercise, healthy food and nutrition*) were the easiest to endorse (item-location estimates of − 1.158 and − 1.135, respectively).

## Discussion

Based on our national random sample (*n* = 900), we found that empirical evidence did not support our null hypothesis associated with the psychometric properties of the HLS-EU-Q47. By excluding poorly fitting items, items displaying DIF and items violating local independence, we succeeded in establishing a psychometrically sound parsimonious 12-item version (HLS-Q12). From a measurement point of view, we found that the HLS-Q12 outperformed other available short versions of the HLS-EU-Q47, such as the HL-SF12 and the HLS-EU-Q16.

### Psychometric properties of the HLS-EU-Q47

#### Overall analyses of HLS-EU-Q47

A 12-dimensional model described the HLS-EU-Q47 data best. This result is perfectly in line with prior research [20, 21]. Hence, applying the HLS-EU-Q47 recommends a complex 12-multidimensional approach returning 12 subscale-scores for each individual. This is of little practical use, especially as proficiency cannot be compared across the different subscales owing to relative points of zero. Owing to few items in each subscale, few of the 12 subscales were sufficiently reliable.

Contrary to common practice when using the HLS-EU-Q47, we cannot recommend reporting either total or health domain subscale scores. The three-dimensional approach, reflecting the three health domains, obtained better data-model fit than the one-dimensional approaches. The three health domain subscales each returned sufficiently large reliability indexes, but lower indexes than those reported by the HLS-EU Consortium [11], Nakayama et al. [18] and Duong et al. [19].

Prior research on people with chronic disease (type 2 diabetes) [20] indicates that the HC subscale brings notable multidimensionality into the data. It is very interesting that this not was supported in our recent analyses of a national sample, because it indicates that the “health care” subscale actually work differently for patients with chronic disease than in the population as a whole. This seems reasonable, as people with chronic disease have more experiences with using health care facilities than the general population.

#### Analyses of HLS-EU-Q47 at item-level

Like previous studies [20, 21] we observed items violating local independence, items displaying DIF, and poorly fitting items. Poorly fitting items which over- or under-discriminate tap into constructs other than the latent trait [64]. Over-discriminating items measure “too much of something else” that is positively correlated with the latent trait and are therefore viewed as less problematic than under-discriminating items. Contrary to Finbråten et al. [20], who found that few items displayed DIF in people with chronic disease (type 2 diabetes), we found several items displaying DIF for age and education, which means that people with higher versus lower levels of education, as well as younger versus older people, probably perceive these items differently. Consequently, comparisons of HL across age groups or educational levels would be invalid [54]. The increased number of items displaying DIF in our study might be expected as the sample of people with type 2 diabetes in Finbråten et al. [20] mainly consisted of elderly persons.

### Psychometric properties of the of HLS-Q12, HL-SF12 and HLS-EU-Q16

#### Overall analyses of the of the HLS-Q12, HL-SF12 and HLS-EU-Q16

Based on the PCA and *t*-test procedures in RUMM, none of the short versions stood out as sufficiently unidimensional. However, considering the lower binominal 95% CI proportions of 0.07, the two 12 item versions HLS-Q12 and HL-SF12 could be considered sufficiently unidimensional [54]. Hagell [41] claims that the width of the binominal 95% CI is influenced by sample size. When investigating dimensionality in a larger sample, binominal 95% CI-proportions might represent acceptable values. Moreover, unidimensionality is a relative matter and depends on the level of precision. Evaluation of unidimensionality should also include an assessment of the purpose of the measurement, together with theoretical and practical considerations [30, 41].

Comparing the one- and three-dimensional approaches to the short versions, the three-dimensional approach obtained lowest deviance and sufficient values for the GOF indexes. Like some previous studies [32, 65] we found that Rasch modelling and CFA returned similar results. Thus, a three-dimensional approach could be recommended for the short versions. However, when applying a three-dimensional approach the three subscales obtained low reliability indexes. The difference in deviance between the one- and three-dimensional approaches was significant; but the differences were rather small for the two 12 item versions.

A one-dimensional approach to the HLS-Q12 and HL-SF12 could probably be defensible as the results point to minor violations of unidimensionality. Further, the one-dimensional approach yields higher reliability indexes than each of the subscales of the three-dimensional approach. Providing one HL score for each individual, the one-dimensional approach to the HLS-Q12 and the HL-SF12 is practical from a clinical point of view. In addition, the HLS-Q12 and HL-SF12 are in line with the conceptual model of Sørensen et al. [16], but dimensionality should be further explored in future studies.

#### Comparing various short versions

Comparing the psychometric properties of the various short versions, the HLS-Q12 obtained best fit to the Rasch model, whereas the HL-SF12 obtained best fit through CFA. The HL-SF12 was found to have several weaknesses, including items with unordered response categories (item 15) and several items displaying DIF (items 15, 26 and 30). Owing to a larger number of items, the HLS-EU-Q16 stood out as more reliable than the other short versions [63]. However, the HLS-EU-Q16 could not be considered sufficiently unidimensional, it had the highest AIC, and did not yield acceptable GOF indexes. In addition, problems regarding item misfit, DIF and unordered response categories were observed.

Altogether, the HLS-Q12 has better psychometric properties than the HL-SF12 and the HLS-EU-Q16. The HLS-Q12 can be deemed the best-targeted scale and free from under-discriminating items and DIF. The HLS-Q12 reflects the conceptual framework, and it is well balanced because it consists of one item from each of the 12 dimensions. The HLS-Q12 version could be well suited for use in HL screenings at both the individual and community levels. Nevertheless, in future studies, we recommend extending the number of response categories from four to six to increase the reliability of the scale [66]. However, item 38 represents a specific concern because this item under-discriminated when applying one-, three- and 12-dimensional approaches to the entire HLS-EU-Q47. In contrast, when we applied a one-dimensional approach to the HLS-Q12, the item showed good model fit. In future validation of the HLS-Q12, this item should be further investigated.

### Item difficulty

Using the HLS-Q12, items 28 (*judge if the information on health risks in the media is reliable*) and 10 (*judge the advantages and disadvantages of different treatment options*) were the hardest items to endorse. Consequently, people may use information that is not evidence-based and potentially harmful to their health. Hence, health professionals should guide individuals in accessing valid and reliable health information. Further, health professionals should help individuals to develop the ability to critically assess health information from different sources. People also need guidance in judging the advantages and disadvantages of various treatment options. Low HL might cause difficulties with promoting and taking responsibility for one’s own health, as well as participating in shared descision-making regarding health issues. According to Nutbeam [67], nurses and other health professionals must become aware of the effects of low HL. Hence, nurses and other health professionals should map HL in their patients and adapt health communications to the individual or target group.

Further research on HL among Norwegians is needed, especially research examining the correlation between HL and health behaviour and the correlation between access to and the use of health services. It would also be interesting to compare HL across cultures and nationalities. However, before comparing HL across nations and cultures, DIF analyses should be performed to investigate the effects of cultural differences on participants’ interpretations of the content of the items.

### Limitations

Ipsos, the agency that performed the sampling and data collection, guarantees representativeness. However, the educational level of this sample was higher than that of the average Norwegian population. In this study, more than half of participants had education at the university level, while this is true of only 33% of the general population [68].

Although around 1000 individuals were recruited from each of the included EU countries, 900 individuals were included in this study. In Rasch modelling, there is no exact recommendations for sample size. However, Linacre [69] recommends 250 individuals for polytomous data and 10 extra individuals per response category. Hair [70] recommends at least 300 individuals when performing CFA. According to these recommendations, a sample size of 900 would be sufficient.

The HLS-Q12 was developed based on the results of Rasch modelling of the HLS-EU-Q47 using two populations, people with type 2 diabetes [20] and the general Norwegian population. However, its further application to other populations will yield more evidence regarding the validity and reliability of the scale. Future validation should be performed using multinational data.

## Conclusions

The HLS-EU-Q47 was found to best fit a 12-dimensional model, which indicates that a multidimensional approach should be applied when the entire HLS-EU-Q47 is used to measure HL. Consequently, it is not statistically defensible to calculate total HL scores for individuals on the basis of the HLS-EU-Q47, and the estimate of HL for a person cannot be derived from his or her raw score on the HLS-EU-Q47. Relying on 12 different but related scale scores for individuals may be impractical from a clinical point of view. Several items on the HLS-EU-Q47 showed misfit, DIF or unordered response categories, which indicates the need to revise the scale. One should be careful in implementing HL actions based on scores obtained via the HLS-EU-Q47. Hence, instruments should be thoroughly validated before being used in large-population surveys.

This study showed that the HLS-EU-Q47 suffers under particular weaknesses. However, our parsimonious HLS-Q12 meets the assumptions and the requirements of objective measurement while still reflecting the conceptual HL model scaffolding the HLS-EU-Q47. Health professionals aiming to adapt their communication to patients’ HL will obviously benefit from a measurement scale like the HLS-Q12 as it could be considered a clinically feasible screening instrument, which does not require advanced on-site psychometric methods. The economic gains that might lie in rationalising health care by applying the short but sufficient HLS-Q12 is relevant for the development of health policies. Researchers aiming to understand which factors impact HL and how HL is related to health outcomes will definitely benefit from an HL scale with sufficient psychometric quality. More importantly, the conclusions from such research have the potential to feed further information back into policymaking.

## Abbreviations

A: accessing health information
AIC: Akaike’s information criterion
ANOVA: analyses of variance
B: understanding health information
BIC: Bayesian information criterion
C: appraising health information
CFA: confirmatory factor analysis
CFI: comparative fit index
CI: confidence interval
D: applying health information
DIF: differential item functioning
DP: disease prevention
ep: estimated parameters
GOF: goodness of fit
HC: healthcare
HL: health literacy
HLS-EU-Q47: European Health Literacy Survey Questionnaire
HP: health promotion
LRT: likelihood ratio test statistics
ML: maximum likelihood estimation
MNSQ: mean square
NNFI: non-normed fit index
PCA: principal component analysis
PRM: polytomous Rasch model
PSI: person separation index
PSR: person separation reliability
RMSEA: root-mean-squared error of approximation
SB scaled χ^2^: Satorra-Bentler scaled chi-square
SRMR: standardised root mean square residual
TLI: Tucker and Lewis fit index
